# Analysis of the Calls Received during the COVID-19 Lockdown by the Mental Health Crisis Helpline Operated by the Professional College of Psychology of Aragon

**DOI:** 10.3390/ijerph19052901

**Published:** 2022-03-02

**Authors:** Alicia Monreal-Bartolomé, Yolanda López-Del-Hoyo, Itxaso Cabrera-Gil, Alejandra Aguilar-Latorre, Marta Puebla-Guedea, Santiago Boira, Jesús Lanero

**Affiliations:** 1 Primary Care Prevention and Health Promotion Research Network, RedIAPP, 28029 Madrid, Spain; aliciamonbart@gmail.com (A.M.-B.); aaguilar@iisaragon.es (A.A.-L.); martapueblag@gmail.com (M.P.-G.); 2 Institute for Health Research Aragón (IIS Aragón), 50009 Zaragoza, Spain; 3 Department of Psychology and Sociology, University of Zaragoza, 50009 Zaragoza, Spain; sboira@unizar.es; 4 Psychology Research Group of the Professional College of Psychology of Aragon (COPPA), 50001 Zaragoza, Spain; itxasocg@gmail.com; 5 Faculty of Health Sciences, San Jorge University, Villanueva de Gállego University Campus, 50830 Villanueva de Gállego, Spain; jesuslanero@cop.es

**Keywords:** mental health, COVID-19, crisis helpline, pandemic, psychological distress, psychological impact

## Abstract

COVID-19 has had a direct impact on the physical and mental health of millions of people worldwide. Therefore, a Mental Health Crisis Helpline (MHCH) was set up and offered free of charge by the Professional College of Psychology of Aragon (COPPA) during the lockdown period. This research aims to study the reasons for the calls, to describe the population segments that used it, and to analyse the possible relationships between the variables studied. A total of 1411 calls were answered and 598 were registered. The main reasons for the calls were: anxiety symptoms, concern for a relative, previous mental health problems, conflicts, and depressive symptoms. Significantly more men called for anxiety symptoms (60.8% vs. 49.5%) versus more women calling regarding a chronic physical illness (3.5% vs. 0.7%), concern about a relative (22.7% vs. 12.4%), care guidelines (6% vs. 1.3%), and bereavement (6.2% vs. 2%). Calls regarding conflict increased slightly as the lockdown period progressed (*p* < 0.001; r = 0.15), in contrast with calls regarding previous psychological conditions and anxiety symptoms (*p* = 0.035; r = −0.09; *p* = 0.005; r = −0.12). These results highlight the intensive use of the MHCH, confirming the need for the implementation of specific psychological care resources in times of crisis.

## 1. Introduction

COVID-19 has spread worldwide and most countries have implemented severe health and social measures to deal with it. As a result, the pandemic that first appeared in China has become a public health emergency of international concern.

Spain declared a state of alarm on 14 March 2020. The drastic measures implemented were to keep people at home, where they had to remain and could only leave in certain situations (for work, to buy basic necessities, etc.). Subsequently, between 30 March and 12 April, all non-essential work activities were suspended, aggravating an already severe economic crisis. By 27 April, a total of 210,773 confirmed cases of COVID-19 had been detected, causing 23,822 deaths, and 102,548 people were reported to have recovered [1,2]. At that time, Spain was the European country with highest infection rate in the world, behind only the United States, and its death toll from the disease was fast approaching that of Italy.

Currently, we can say that the virus has had a direct impact on the physical health of millions of people and, what is more, represents a major threat to mental health worldwide. In fact, two studies of COVID-19 patients found a high level of post-traumatic stress disorder (PTSD) symptoms (96.2%) and a significantly higher level of depressive symptoms (29.2%; *p* = 0.016) [3,4]. Other studies with patients with pre-existing psychiatric disorders reported a worsening of psychiatric symptoms [5,6]. On the other hand, we found studies investigating health care workers that reported an increase in depression and symptoms of depression, anxiety, psychological distress, and poor sleep quality [7,8,9,10,11,12,13,14,15,16]. As for studies in the general population, these revealed lower scores for psychological well-being and higher scores for anxiety and depression compared to the period pre-COVID-19, while there were no differences when comparing these symptoms during the initial phase of the outbreak with a follow-up made four weeks later [16,17,18,19]. A study on the short-term psychological impact of the pandemic in the Spanish population [20] revealed the presence of depression, anxiety, and post-traumatic stress disorder. A variety of factors were associated with an increased risk of psychiatric symptoms and/or low psychological well-being, including female gender and the poor health of family members and relatives with COVID-19 [21].

Thus, the psychological consequences of this crisis have been manifold, with the presence of anxiety, depression, post-traumatic stress disorder, or insomnia in a significant percentage of the population [22,23,24,25,26,27]. The outbreak of COVID-19 is therefore an emotional challenge for everyone, and especially for people who are already at risk (e.g., those suffering from depression).

However, the mental health resources of Spain’s National Health System (NHS) were clearly shown to be insufficient. According to the 2020 report of the Spanish Government Ombudsman, based on 2018 data, the average number of psychologists in our NHS is 6 per 100,000 inhabitants [27], a figure well below the European Union average (18 psychologists per 100,000 inhabitants).

For this reason, and in anticipation of the psychological effects of the pandemic and the state of alarm on the population, a crisis helpline offered by the Professional College of Psychology of Aragon (COPPA, by its Spanish acronym) was set up during the period in which the entire population of the Aragon region was in lockdown. In order to provide a comprehensive response to COVID-19, traditional face-to-face intervention had to be replaced by new forms of remote therapy, both online [22,28] and by telephone [29]. Helplines have traditionally played a crucial role in crisis situations [30,31,32,33] and this was a natural course of action in the pandemic [29,34,35,36]. In fact, practice in China indicates that online mental health services cannot replace crisis helplines. Many people still have limited access to computer- or Internet-based services, do not know how to use them, and do not possess a smartphone [29].

This research aims to study the reasons that led to the calls made to the helpline set up for the psychological care of the population of Aragon during the state of alarm, during which stay-at-home, self-isolation, and quarantine measures were put in place, lasting from March to the end of May 2020. It also aims to describe the type of population that used this service and the main events that occurred during this period in Spain, as well as to analyse the possible relationships between the different variables studied.

## 2. Materials and Methods

### 2.1. Implementation of the Mental Health Crisis Helpline for the Public

On 23 March, COPPA, together with the Department of Health of Aragon and with collaboration from the Rey Ardid Foundation, launched a free helpline through which different healthcare professionals dealt with queries from the Aragonese population.

By calling 876 036 778 between 8 a.m. and 8 p.m. from Monday to Friday, patients accessed psychological care provided by more than 250 professionals at a time of maximum uncertainty. The existence of this telephone number was publicised by both local media and social media. Thanks to these volunteers, some 30 calls a day were answered.

In order to set up this helpline, COPPA contacted psychology professionals on 18 March via email, requesting their collaboration for the Mental Health Crisis Helpline (MHCH). Volunteer psychologists contacted COPPA, indicated their availability for the service, and supplied their mobile phone number. These mobile numbers were given to the Department of Health to allow calls to be transferred to them via a switchboard. Therefore, the users had knowledge of the switchboard number that was provided when the service was publicised by the different administrations and entities. The service operated from Monday to Friday and three 4-h shifts were established: 8 a.m.–12 p.m., 12–4 p.m., and 4–8 p.m.

At the end of a call, each volunteer psychologist filled in a call log, which was periodically requested by COPPA.

### 2.2. Population

This telephone counselling and support service was accessible to anyone in the autonomous community of Aragon, Spain, who called the contact telephone number. Because health professionals had access to other telephone support services, most of the users of the service were from the general population, not healthcare workers. There was no age limit for users of this helpline, although the procedure for dealing with minors was to speak to the parent or guardian first, before attending to the child when required.

### 2.3. Intervention

The volunteers were COPPA-certified psychologists who were given intervention guidelines based on those used by the Psychological Intervention Group for Emergencies and Catastrophes (GIPEC), within COPPA, for dealing with calls. They were also sent material with recommendations for intervention provided by the Professional College of Psychology of Navarre [37], in addition to a series of practical indications on how the service works.

The types of assistance provided by the helpline were: (a) analysis of the reason for the call; (b) identification of symptoms; (c) direct intervention with the user; and (d) referral to specialised psychiatric, psychological, medical or social services where required. Not only psychological support was provided: when referral to other social and health services was required, in certain cases, the professionals provided information on different resources (social services, mental health services, local councils, etc.) to facilitate users’ access to those services.

### 2.4. Winding-Down of the Service

On 23 April 2020, Spain’s central government, which had taken over the functions of the regional governments, announced the implementation of lockdown de-escalation measures for the population, and children under the age of 14 years were allowed to leave home between 9 a.m. and 9 p.m. The lifting of the lockdown, referred to as de-escalation, was implemented in four stages, with restrictions in the country progressively easing by stage as the number of cases decreased. On 17 May, for the first time since the state of alarm was declared on 14 March, fewer than 100 deaths per day were reported. A few days later, there were regions that did not report any cases. Because of this, and with the situation also under control in Aragon, COPPA and the government of Aragon decided that it was no longer necessary to continue to provide the helpline. Consequently, on 29 May, the MHCH was wound down and the service it offered to the population came to an end. However, the possibility remained that it could be reinstated were the need to arise again.

### 2.5. Data Collection

The data were collected in two ways: from the telephone calls made and the date of the calls; and from the register completed by the psychologists who received the calls and reported the following pre-established variables through a form using a website. The information was compiled by the COPPA data centre as follows:

- Call date
- Caller gender
- Caller age group
- Main reason
- Relevant free text in relation to the call, which was coded as other reasons or other relevant information
- Response given

### 2.6. Data Analysis

We performed a descriptive analysis of the characteristics of the participants in the whole sample using means, standard deviation (SD), frequencies, percentages, and the χ2 statistic to assess possible relationships between the sociodemographic variables (gender and age range) and the other variables of interest.

Thus, we explored whether there was dependence between the variables reflecting the different reasons for calling found (general non-main reasons) and the sociodemographic variables (gender and age), using the χ2 statistic. Thus, variables were not considered to be independent when *p*-value < 0.05, provided that no more than 20% of calls had a frequency equal to or less than 5 (when this last condition was not met, we used Fisher’s exact test, whose *p*-value also had to be lower than 0.05).

In addition, the correlations between the dates of the calls and the different variables (gender, age and general motives) were analysed using Spearman’s correlation coefficient. In all cases, statistical significance corresponded to *p*-value < 0.05.

The data analysis was performed using the IBM Statistical Package for the Social Sciences (SPSS) version 25 [38]. 

## 3. Results

**Figure 1:**
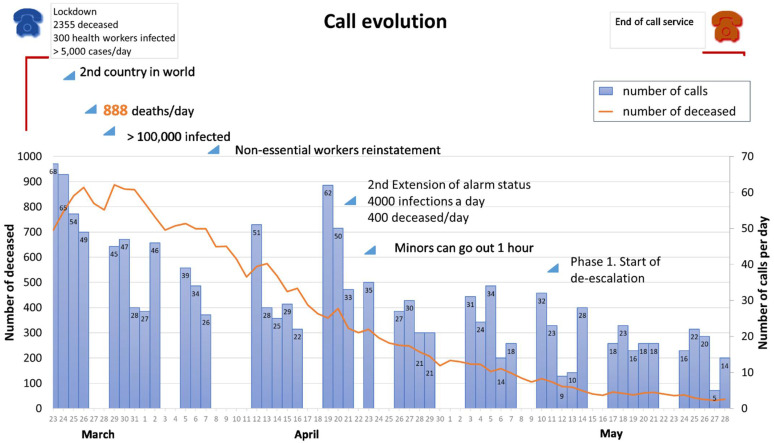
Evolution of the number of calls and deaths according to the main milestones of the pandemic.

As of 23 March, when the MHCH began operating in Aragon, a total of 1411 calls were answered in the 46 days that the service was active, with an average of 30.67 calls/day (SD = 15.34). The majority of these were made during the first 5 weeks of the service (65.13% of calls, despite the fact that the service was active on fewer days during those weeks, owing to public holidays). Those calls were dealt with by more than 250 volunteer psychology professionals who were members of COPPA, and 598 of the total number of calls received were registered. Figure 1 shows the evolution in the number of calls over the different weeks and the main events and milestones that occurred during the period the helpline was active.

It should be noted that some of these calls were recurring (14.55%). In addition, six calls were taken from autonomous communities other than Aragon (namely Madrid, Catalonia, the Canary Islands, and the Balearic Islands), and even from abroad. There were also three erroneous calls, which were seeking medical support rather than psychological support, and one was a call meant for 112 (emergency services). Another call was received requesting medical assistance, while another caller mistook the helpline for the COVID-19 health assistance line operated by the regional health service.

Finally, 73.91% of the registered calls were made by women, compared to 26.09% made by men. Moreover, the majority of the calls were made by adults (77.9%), compared to older people (19.1%) and young people/children (3%).

### 3.1. Reasons for the Calls

The main reason prompting calls to the helpline was emotional distress due to anxiety-type symptoms (38%): excessive worry or obsession, anguish, stress, uncertainty, phobia, burnout, panic attacks, difficulty concentrating. This was followed by concern for a relative or friend (16%), a category initially included in anxiety that was finally removed due to its high weighting; the category of fear (of being infected or of infecting) (8%) was likewise removed from anxiety.

Another category was depression, which, as its name suggests, includes depressive symptoms (4%): sadness, uncontrolled crying, lack of motivation, apathy, guilt, hopelessness, catastrophic thinking, suicidal ideation; in some cases, anxiety co-existed with depression (3%), and, in some cases, there was even great emotional lability.

**Figure 2:**
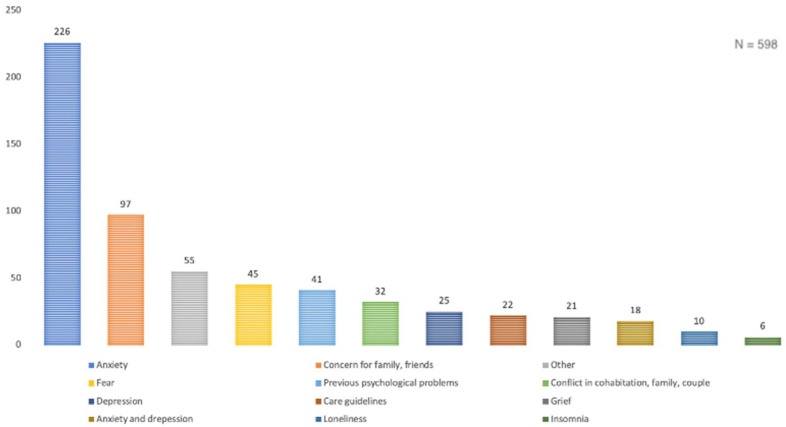
Distribution of the main reasons for calling the COPPA MHCH.

Another of the most common reasons for calling was a previous history of the situation or prior psychological problems aggravated by the situation of confinement (7%), among which the majority had suffered from anxiety, depression, obsessive-compulsive disorder (OCD), severe mental disorders (SMD), and alcohol and drug consumption. There were also many calls describing conflicts within families, relationships or cohabiting individuals as the main reason (5%), as well as bereavement due to the recent death of a family member or somebody very close (4%). Likewise, numerous calls were received requesting guidelines for the care of a sick relative or minor with depression, attention-deficit/hyperactivity disorder (ADHD), anxiety, dementia, behavioural problems, etc., and for explaining the situation to younger children (4%). Calls were also received for isolated problems, such as loneliness (2%) and insomnia (1%). Finally, the category “other” included calls for less frequent aspects, such as guilt, difficulty in decision-making, emotional dependence, relief, disorientation, erroneous calls, no reason given, ruminations, suicidal risk, discomfort, requests for information from the service, etc. Figure 2 shows the main reasons for calling.

In many cases, the main reason for calling was not the only one. In addition to the main reason, there were other secondary reasons, such as loneliness, shame, guilt, irritability, discomfort due to a stressor other than COVID-19 (relationship breakdown, family and/or cohabitation conflicts, illness of a family member, redundancy), obsessions, and somatisation. Table 1 shows the percentages of calls according to main reasons, as well as the most common reasons regardless of whether these were main or secondary.

The causes behind the reasons for the calls were very diverse, including economic problems; work problems, due to fear of becoming infected at work (some of which were from individuals working in healthcare, mostly in nursing homes), burnout, conflicts with colleagues, etc.; family problems, due to concern for a relative (because they lived alone or in a nursing home, because they could not see them, because they had COVID, out of fear of being infected by or of infecting them, etc.), cohabitation conflicts, negative relationships, etc.; social problems, due to conflict with friends, the impossibility of making plans because the callers were at risk, loneliness, lack of support, relatives living far away, etc.

Finally, we would like to highlight the fact that we received five calls (0.8%) in which the caller or someone close to them presented suicidal ideation or the intention or imminent risk of carrying out the action. We would also like to note that there were 36 calls (6%) from people who felt lonely.

### 3.2. Variable Dependency Analysis

Statistically significant differences were observed in terms of anxiety and gender, with more men calling about anxiety symptoms (60.8% vs. 49.5% of women). By contrast, significantly more women called for a chronic physical condition (3.5% vs. 0.7% in men), for concern about a relative or friend (22.7% vs. 12.4% in men), for care-giving guidelines (6% vs. 1.3% in men), and for bereavement (6.2% vs. 2%). A certain tendency was observed in the conflict variables, with more women calling about this, and fear, with a slightly higher number of calls from men (Table 2).

No statistically significant associations were observed between age ranges and the different reasons for calling. However, there was a certain trend in the variables of anxiety (*p* = 0.072)—with a lower percentage of older people calling for anxiety—followed by young people and children and, finally, adults, and insomnia (*p* = 0.069), with more calls by young people and children, followed by older people and adults (Table 3).

### 3.3. Correlations

After correlational analysis, we can observe how calls due to conflict (family, relationship, etc.) increased slightly as lockdown progressed (*p* < 0.001; *r* = 0.15), and how the opposite occurred with calls made by people with prior conditions, who called more at the beginning of the lockdown period (*p* = 0.035; *r* = −0.09). Calls owing to anxiety or anxiety symptoms also decreased slightly over time (*p* = 0.005; *r* = −0.12). In this sense, it can be observed that older people increased their calls to the helpline as lockdown progressed, and younger people called more towards the beginning (*p* < 0.001; *r* = 0.16) (Table 4).

There were also numerous statistically significant correlations between the other variables studied, but these are considered to be of less interest for the purposes of this study, so they are not described here.

## 4. Discussion

The results of the present study highlight the intensive use made of the MHCH launched by COPPA together with the Department of Health of Aragon, and with collaboration from the Rey Ardid Foundation, during the most difficult months of the pandemic in Aragon. This aspect emphasizes the great need for psychological care by the population of the region. Specifically, while the helpline was active, 1411 calls were handled, and the number of lost calls was 238. Therefore, with a total of 1649 calls, the percentage level of attention was 86%. In total, 598 were registered for detailed analysis. These figures, together with those of other studies in Spain [36,39] and other countries [29,40], reflect the usefulness of this type of service (helplines) during pandemics.

In terms of the socio-demographic analysis of the data, women used the service almost three times more than men (73.91%), which is consistent with the results of other studies [16,29,36,38,40]. This may have been due to the fact that lockdown led to difficult situations for families, in which women were overburdened with responsibilities [36].

With regard to age, by far the most prevalent age range was adults (77.9%), compared to older people (19.1%) and children and young people (3%), which coincides with similar studies carried out in Spain [36,38]. However, other preliminary studies on the immediate psychological impact of the pandemic showed that young people were the most affected [20,41]. In particular, a specific study on telephone counselling in Portugal shows that they were the ones who used this type of assistance the most [35]. This discrepancy between the present study and the aforementioned studies could be explained by different reasons. Firstly, the higher participation of young people in the aforementioned study by Ribeiro et al. [35] could have been due to the fact that they reflected data from services aimed primarily at the university population. With regard to the two prevalence studies carried out in Spain, it is important to highlight that these studies made use of online survey platforms and were disseminated through social media, which are more accessible and known to young people. Finally, the studies by Berdullas et al. [36] and the Colegio Oficial de la Psicología de Castilla-La Mancha [39] coincide closely in their descriptive results because they share methodological characteristics. Those studies analysed data from crisis counselling helplines that were aimed at the general population and disseminated through similar official bodies (the Spanish Ministry of Health, the General Council of Psychology of Spain and the regional Professional Colleges of Psychology). Moreover, these data could be considered a strength of the MHCH due to the high participation of older people, a population group particularly impacted by the pandemic, and could be consistent with studies showing that older people regularly prefer telephone resources over other types of resources, such as videoconferencing [42].

On the other hand, with regard to the evolution of the calls, it is worth noting that the two days on which the helpline received the most calls were 24 and 25 March, which coincided with one of the most dramatic moments of the pandemic situation in Spain. On the 24th, special concern was expressed about the situation in nursing homes throughout the country; 13% of healthcare workers were infected and the central government admitted that there was a lack of material with which to deal adequately with the pandemic. On the 25th, Spain overtook China in number of deaths and became the second country, after Italy, on the list of countries most affected by the pandemic. The high number of calls continued during the first two weeks, in line with the beginning of the rise in the number of deaths and daily infections, with figures close to 1000 deaths/day and more than 100,000 infections/day. The third day with the highest number of calls over the entire period in which the MHCH was active was Monday 20 April, which coincided with the mass application of testing and an increase in the number of infected people. In addition, the extension of the state of alert for a further 15 days was still on the table, given that the situation remained critical. That week produced the third-highest number of calls (180 calls), despite including a public holiday, on which the service was not active, concurring with the extension of the state of alarm declared by the Spanish government on 24 April and with the possibility of minors being able to leave home for 1 h a day with restrictions. Finally, the fourth week with the highest number of calls was the week of 13–19 April, when there was a peak in the number of deaths/day (500). The MHCH was closed over the Easter period (9 and 10 April).

It should therefore be noted that throughout the time that the MHCH was active, there was an increase in the number of calls related to information about the situation provided by the media (number of deaths, infection rate, etc.) and to the expectations that this information produced (possible extensions of the state of alarm, de-escalation, etc.), which highlights the great importance of communication management in this type of crisis situation. Finally, the decrease in the number of calls towards the end of the period in which the helpline was active may have been due not only to the improvement in the situation and the beginning of lockdown being lifted in the country, but also to the possible habituation to the situation by part of the population (over time we became accustomed to hearing about very large numbers of deaths), as well as to the change in the way these data were reported (infections/100,000 inhabitants, deaths/100,000 inhabitants).

In relation to the reasons for consultations/calls, consistent with what has been found in previous literature [36,38,40,43,44], the presence of anxiety symptomatology was the main concern of callers (38% as the main reason and 52.4% as a reason in general). In relation to these symptoms, we can also highlight that concern for family or friends (16–20.1%), fear of infection (8–16.4%) and insomnia (1–5.6%), depressive symptoms (4–23.1%), calls due to prior psychological condition (7–11.7%), bereavement symptoms (4–5.1%), and loneliness (2–6.6%) were also very common. Other more prominent reasons were conflicts in situations of cohabitation, families and relationships (5–18.5%), as well as requests for information or care guidelines (4–4.7%).

Finally, significantly more men called for anxiety symptoms (60.8% vs. 49.5% of women). By contrast, significantly more women called about a chronic physical condition (3.5% vs. 0.7% in men), concern for a relative or friend (22.7% vs. 12.4% in men), care-giving guidelines (6% vs. 1.3% in men), and bereavement (6.2% vs. 2%). The calls regarding both concern about family members and conflict may have been related to the above-mentioned increase or overload in women’s responsibilities, and to the fact that women seemed to be experiencing more gender-based violence in this situation of crisis [45].

Furthermore, calls regarding conflict increased slightly as time in lockdown progressed (*p* < 0.001; *r* = 0.147), with the opposite being found for calls regarding anxiety symptoms, which decreased as time passed (*p* = 0.005; *r* = −0.117). This trend is consistent with the study by Li et al. [43], who observed how emotional symptoms, such as anxiety and depression, decreased over the time that their psychological helpline was active in Wuhan, while the demand for practical problem-solving during the pandemic gradually increased. Calls made by people with prior conditions also decreased, with these types of calls being more prevalent at the beginning of lockdown (*p* = 0.035; *r* = −0.087). However, older people were observed to increase their calls to the service in line with time spent in lockdown, and younger people called more towards the beginning (*p* < 0.001; *r* = 0.155).

### Strengths and Limitations

The strengths of this study are that it offers a vision of the consultations of the Aragonese population during the state of alarm, which lasted from March to the end of May 2020. Furthermore, the reasons that motivated the calls to the helpline enabled the psychological reasons to be analysed. Likewise, the type of population that used this service and the main events that occurred during this period in Spain were described, and the possible relationships between the different variables studied were analyzed. The limitations of the study were that not all the calls could be answered and that not all the calls answered were registered, since their registration was something that the psychologists offered to do voluntarily. Further research is needed regarding the scripts and qualitative data obtained in order to give the article theoretical value [46].

## 5. Conclusions

The characteristics of the psychological conditions of individuals and their focus of attention during the pandemic changed over time, which deserves attention, and psychological intervention measures should also adjust the focus of attention accordingly.

In summary, the data provided in this study highlight the need for specific psychological care resources, such as the MHCH, to be put in place to meet the psychological needs of the population in times of crisis, such as during this pandemic, and with particular emphasis given to the psychological health of the most vulnerable groups.

## Figures and Tables

**Table 1:** Reasons for the calls.

	Main Reasons (%)	General Reasons (%)
Anxiety symptomatology	38%	52.4%
Concern for family, friends	16%	20.1%
Other	9%	-
Fear	8%	16.4%
Prior condition	7%	11.7%
Conflicts in cohabitation, family, relationship	5%	18.5%
Depressive symptomatology	4%	23.1%
Bereavement	4%	5.1%
Care guidelines	4%	4.7%
Anxiety and depression symptomatology	3%	-
Loneliness	2%	6.6%
Insomnia	1%	5.6%
Chronic physical condition	-	2.7%

**Table 2:** Cross-tabulation of variables with gender.

Correlation between Variables	Females	Males	*p*-Value	Chi-Squared
Bereavement	6.2%	2%	0.039	4.25
Conflicts	20.3%	13.7%	0.071	3.25
Prior psychological conditions	12%	11.1%	0.767	0.09
Chronic physical condition	3.5%	0.7%	0.049	3.36
Anxiety symptomatology	49.5%	60.8%	0.017	5.74
Depression symptomatology	22.4%	24.8%	0.530	0.40
Concern for a family member/friend	22.7%	12.4%	0.006	7.48
Care-giving guidelines	6%	1.3%	0.020	5.42
Loneliness	6.4%	7.2%	0.743	0.11
Fear	14.7%	20.9%	0.072	3.23
Insomnia	4.8%	7.2%	0.265	1.24

**Table 3:** Cross-tabulation of variables with age ranges.

Correlation between Variables	Children and Youths	Adults	Older People	*p*-Value	Chi-Squared
Anxiety symptomatology	50%	54.9%	42.9%	0.071	5.25
Insomnia	16.7%	4.6%	7.1%	0.069	5.26

**Table 4:** Cross-tabulation of variables with date of call.

Correlation between Variables	*p*-Value	r
Gender	0.574	−0.023
Age	*p* < 0.001	0.155
Bereavement	0.242	0.049
Conflicts	*p* < 0.001	0.147
Prior psychological conditions	0.035	−0.087
Physical illness	0.183	−0.055
Anxiety symptomatology	0.005	−0.117
Depression symptomatology	0.936	0.003
Concern for a family member/friend	0.084	−0.071
Care-giving guidelines	0.113	0.066
Loneliness	0.349	−0.039
Fear	0.169	0.057
Insomnia	0.972	0.001

## Data Availability

The data presented in this study are available on request from the corresponding author.
